# Systematic Evaluation of Key L-Carnitine Homeostasis Mechanisms during Postnatal Development in Rat

**DOI:** 10.1186/1743-7075-9-66

**Published:** 2012-07-17

**Authors:** Binbing Ling, Caroline Aziz, Jane Alcorn

**Affiliations:** 1 College of Pharmacy and Nutrition, University of Saskatchewan, 110 Science Place, Saskatoon, SK, S7N 5C9, Canada; 2 Toxicology Centre, University of Saskatchewan, 44 Campus Drive, Saskatoon, SK, S7N 5B3, Canada

**Keywords:** L-Carnitine, Homeostasis, Postnatal development, Rat

## Abstract

**Background:**

The conditionally essential nutrient, L-carnitine, plays a critical role in a number of physiological processes vital to normal neonatal growth and development. We conducted a systematic evaluation of the developmental changes in key L-carnitine homeostasis mechanisms in the postnatal rat to better understand the interrelationship between these pathways and their correlation to ontogenic changes in L-carnitine levels during postnatal development.

**Methods:**

mRNA expression of heart, kidney and intestinal L-carnitine transporters, liver γ-butyrobetaine hydroxylase (Bbh) and trimethyllysine hydroxylase (Tmlh), and heart carnitine palmitoyltransferase (Cpt) were measured using quantitative RT-PCR. L-Carnitine levels were determined by HPLC-UV. Cpt and Bbh activity were measured by a spectrophotometric method and HPLC, respectively.

**Results:**

Serum and heart L-carnitine levels increased with postnatal development. Increases in serum L-carnitine correlated significantly with postnatal increases in renal organic cation/carnitine transporter 2 (Octn2) expression, and was further matched by postnatal increases in intestinal Octn1 expression and hepatic γ-Bbh activity. Postnatal increases in heart L-carnitine levels were significantly correlated to postnatal increases in heart Octn2 expression. Although cardiac high energy phosphate substrate levels remained constant through postnatal development, creatine showed developmental increases with advancing neonatal age. mRNA levels of Cpt1b and Cpt2 significantly increased at postnatal day 20, which was not accompanied by a similar increase in activity.

**Conclusions:**

Several L-carnitine homeostasis pathways underwent significant ontogenesis during postnatal development in the rat. This information will facilitate future studies on factors affecting the developmental maturation of L-carnitine homeostasis mechanisms and how such factors might affect growth and development.

## Introduction

L-Carnitine is a conditionally essential nutrient that functions in a number of physiological processes vital to normal neonatal growth and development [1]. With transition to extrauterine life the carbohydrate rich, low-fat umbilical blood supply is replaced by the high fat, low glucose diet of the breast milk. This switch in nutrition source requires rapid physiological adaptations in the newborn to enhance gluconeogenic processes and fatty acid oxidation pathways to meet the energy demands of developing neonate. L-Carnitine plays an important role in the enhancement of fatty acid utilization during this adaptive period and throughout postnatal development [1]. By regulating the movement of long chain fatty acids into mitochondria and making them available for β-oxidation, L-carnitine has an obligatory function in cellular energy production. Furthermore, L-carnitine has a role in the removal of toxic fatty acyl-CoA metabolites from the mitochondria to maintain an appropriate balance between free carnitine and its acylated forms [2]. This vital role in cellular metabolism identifies L-carnitine as essential to the maintenance of normal mitochondrial function and in the prevention of disease [2]. Any disruption in the L-carnitine homeostatic processes during postnatal maturation might adversely impact the developing and growing neonate [3].

L-Carnitine homeostasis represents a balance between 1) *de novo* biosynthesis, 2) intestinal absorption from dietary sources, 3) uptake and release by the tissues, and 4) renal reabsorption [4]. In early postnatal life, L-carnitine biosynthesis is limited as a result of immature γ-butyrobetaine hydroxylase activity (Bbh) (approximately 10-12 % adult levels), the enzyme that mediates the last step in L-carnitine biosynthesis [5-7], but not trimethyllysine hydroxylase (Tmlh) activity, the first enzyme in L-carnitine biosynthesis [6]. The activity of Bbh increases with age and reaches adult values later in postnatal development [6,8]. Delayed Bbh maturation suggests a reliance on dietary L-carnitine sources particularly during the immediate postnatal period.

Dietary L-carnitine is absorbed across the gastrointestinal mucosa by active transport systems at the luminal enterocytic membrane [4]. Membrane transporters, in particular the organic cation/carnitine transporters (Octn1, Octn2, and Octn3), also mediate the tissue distribution and renal reabsorption of L-carnitine and thus play a critical role in L-carnitine homeostasis [9-11]. Of these Octn transporters, Octn2 is the major high affinity sodium-dependent L-carnitine transporter [12,13] that plays a major role in regulating plasma and tissue pools of L-carnitine. Octn transporters might undergo changes in expression with postnatal maturation, but limited data are available on their ontogeny in different tissues [12,14,15].

As major components of the L-carnitine shuttle system, L-carnitine acyltransferase enzyme (Cpt) systems play important roles in fatty acid metabolism and energy production and maintaining an appropriate balance between free and acylated fatty acids [16]. Cpt1 on the mitochondrial outer membrane catalyses the first step of mitochondrial import of long chain fatty acids by converting them from fatty acyl-CoA to acylcarnitines [17]. On the inner mitochondrial membrane Cpt2 reconverts the acylcarnitines to the respective CoA esters releasing free L-carnitine and making fatty acids available for β-oxidation [18]. Limited information is also available on the ontogeny of Cpt enzymes.

Given our limited understanding of the ontogenesis of L-carnitine homeostasis pathways, we conducted a systematic evaluation of the developmental changes in tissue L-carnitine levels, tissue Octn mRNA expression and immunohistochemistry, liver Bbh expression and activity, heart Cpt1b and Cpt2 expression and activity, and heart high energy phosphate compounds at different postnatal ages with consideration of the known maturation of L-carnitine biosynthesis for the rat [19]. At each postnatal age, these evaluations were conducted in tissues sampled from the same animal to avoid the expected interindividual variability and replicated such that the sample size was four animals per postnatal age group. Such a systematic evaluation is important for investigations into environmental and pathophysiological factors that might impact the normal maturation of these processes and the long-term impact on health and risk for chronic disease, which is a principal objective of our laboratory.

## Material and Methods

### Animals and Chemicals

Female Sprague–Dawley rats ordered at different gestation stages were obtained from Charles River Canada (St. Constant, PQ) and were housed singly in a temperature and humidity controlled facility (22 °C ± 2 °C) on a 12-hour light:dark cycle (0700 h – 1900 h). All rats were allowed a 7-day acclimatization period and had free access to food (Prolab® RMH 3000, Purina, Inc., Richmond, IN) and water throughout the study. The dams were closely monitored near parturition to identify the exact time of birth. At birth, the litter size was equalized to 10 pups per dam. The dam was considered the experimental unit (n = 6) and blood and tissues were pooled from 5 pups from each dam to carry out the various analyses. Rat pups at postnatal day (PD) 4, 8, 11 and 20 were anaesthetized with isoflurane, and blood (200 – 500 μL depending on age) was collected by intracardiac puncture. The rats were immediately sacrificed and heart, intestine, kidney, and liver were rapidly excised and flash-frozen in liquid nitrogen with storage at −80 °C until analysis. All procedures were conducted in accordance with the Canadian Council of Animal Care guidelines for the care and use of laboratory animals and were approved by the Animal Research Ethics Board of the University of Saskatchewan.

RNeasy Midi kits were obtained from Qiagen Inc. (Mississauga, ON). The QuantiTect SYBR Green RT-PCR kit was from Applied Biosystems (Foster City, CA). The Advanced Protein Assay kit was obtained from Fluka (Buchs, Switzerland). L-Carnitine and other chemicals not otherwise specified were obtained from Sigma-Aldrich (St. Louis, MO) and were the highest purity grade available.

### L-Carnitine Analysis in Serum and Heart

Serum and heart L-carnitine was quantified by HPLC-UV with pre-column derivatization according to Feng et al. [20]. Briefly, the pooled heart samples were homogenized with phosphate buffer (50 mM, pH 7.4) in a ratio of 50 mg tissue:250 μL buffer. The homogenate was centrifuged at 2500 × *g* for 10 min at 4 °C. The supernatant or serum (20 μL) sample was precipitated using acetonitrile and methanol (9:1 v/v). A 300 mg mixture of Na_2_HPO4 and Ag_2_O (9:1 wt/wt) and 300 mg of KH_2_PO4 were added followed by a 1 h vortex mixing. Derivatization reagent (40 mg/mL ρ-bromophenacyl bromide with 50 μL 40 % tetrabutylammonium hydroxide solution) was added into the organic extract. The reaction mixture was incubated at 60 °C for 2 h followed by centrifugation at 12,000 × *g* for 15 min. L-Carnitine was analyzed using a Hewlett Packard 1050 HPLC system with Diode Array Detector, Quaternary Pump and Autosampler. Samples (10 μL) were injected onto a CN (cyano) column (HyperClone 5 μm, 250 × 4.6 mm, Phenomenex, Torrance, CA) with detection wavelength set at 260 nm. The mobile phase (90 % acetonitrile/10 mM citric-phosphate buffer, pH 3) was delivered at a flow rate of 1 mL/min. The standard curve range was linear (r^2^ >0.99) between 2.5-40 μmol/L. Intra- and interassay accuracy and precision ranged from 6 %-14 %.

### Heart Carnitine Palmitoyltransferase (Cpt) Enzyme Activities

Heart Cpt enzyme activities were measured using the spectrophotometric method described by Bieber et al. [21]. Briefly, frozen heart tissue was homogenized in 10 % (wt/v) homogenization buffer (20 mM HEPES, 140 mM KCl, 10 mM EDTA and 5 mM MgCl_2_, pH 7.4) supplemented with 3 mg nagarse using Polytron homogenizer (Brinkmann Instruments, Rexdale, Canada). The homogenate was then centrifuged at 500×*g* for 10 min at 4 °C. The supernatant was collected in new tubes and centrifuged at 9000×*g* for 35 min at 4 °C. The pellet was then washed with the homogenization buffer without nagarse and centrifuged at 9000×*g* for 35 min at 4 °C. The washed pellet was resuspended in 200 μL homogenization buffer without nagarse. Protein concentrations were measured using the Advanced Protein Assay kit with bovine serum albumin as standard. The optimal protein concentration and reaction time to give linear product formation were initially determined. To determine total Cpt activity 20 μg protein was assayed in 200 μL mL reaction buffer containing 20 mM HEPES, 1 mM EGTA, 220 mM sucrose, 40 mM KCl, 0.1 mM 5,5’-dithio-bis (2-nitrobenzoic acid) (DTNB), 1.3 mg/mL BSA, and 40 μM palmitoyl-CoA, pH 7.4. The reactions were initiated by adding 1 mM L-carnitine and read at 412 nm after 5 min incubation at 37 °C using Synergy HT Multi-Mode Microplate Reader (Biotek instrument, USA). Cpt2 activity was determined using the same reaction conditions as total Cpt except 10 μL (0.2 mM) Cpt1 inhibitor, malonyl-CoA, was added into 200 μL of the reaction mixtures to obtain a final concentration of 10 μM. Cpt1 activity was calculated by subtracting the Cpt2 activity from the total Cpt activity. The Cpt activity was calculated as amount of CoASH released per min per mg protein, which is based on the 5-thio-2-nitrobenzoate formation from CoASH-DNTB reaction. The extinction coefficient for 5-thio-2-nitrobenzoate was 13.6 mM/cm and was used to calculate enzyme activity [22].

### Liver γ-Butyrobetaine Hydroxylase (Bbh) Enzyme Activity

The liver tissue was homogenized in homogenization buffer consisting of 300 mM sucrose, 1 mM EGTA, and 50 mM Tris, pH 7.5 using 1:4 mass to volume ratio. The homogenate was then centrifuged at 13,000×*g* for 30 min at 4 °C. The supernatant was collected and centrifuged at 100,000×g for 1 h at 4 °C. 300 μL of the supernatant representing the cytosolic fraction containing Bbh was then transferred into a dialysis tubing cellulose membrane (molecular weight cutoff of 12,000 Da, D9777, Sigma, USA), and submerged in 5 L dialysis buffer (75 mM KCl, 0.1 mM DTT and 0.5 mM EDTA in sodium phosphate buffer, pH 7.4) overnight at 4 °C. The dialyzed sample (20 μL) was tested for L-carnitine residue and the remaining dialysate was stored at −80 °C for Bbh testing. The optimal protein concentration, substrate concentration and reaction time to give linear product formation was determined for each age group. For determination of Bbh activity in rat pup liver, 1 mL reaction buffer consisting of 0.2 mM γ-butyrobetaine, 20 mM potassium chloride, 3 mM 2-oxoglutarate, 10 mM sodium ascorbate, 0.4 mg/mL catalase in 20 mM potassium phosphate buffer pH 7.0 was prepared. The reaction was initiated by adding 2 μL ferrous ammonium sulfate [23] (final concentration: 0.25 mM) and 20 μL dialyzed enzyme into 78 μL reaction buffer and incubated for 25 min at 37 °C using a Boekel/Grant Orbital and Reciprocating Water Bath (Model ORS200, Expotech, USA). The reaction was then terminated by adding 10 times the volume of acetonitrile:methanol (9:1). The mixture was centrifuged at 13,000×*g* for 2 min and the supernatant was used for L-carnitine analysis by HPLC.

### Total mRNA Isolation and Quantitative RT-PCR Analysis

Total mRNA was extracted from different tissues using Qiagen RNeasy Midi Kits according to manufacturer instructions. RNA purity and quantity were determined spectrophotometrically by measurement at 260 nm and the OD_260_/OD_280_ ratio, respectively, with a Synergy HT Multi-Mode Microplate Reader (Biotek instrument, USA). Total RNA was stored at −80 °C until analysis. Gene sequences were obtained from the National Center for Biotechnology Information GeneBank and specific primers were designed using Primer3 software (Whitehead Institute for Medical Research. Primer 3: http://frodo.wi.mit.edu/primer3/) (Table 1). Quantitative RT-PCR (QRT-PCR) analysis was carried out using a QuantiTect SYBR Green RT-PCR kit and an Applied Biosystems 7300 Real-Time PCR system. The QRT-PCR protocol was carried out according to manufacturer’s instructions. The protocol consisted of reverse transcription (1 cycle at 48 °C, 30 minutes), PCR initial activation step (1 cycle at 95 °C, 15 minutes), three-step thermal-cycling (40 cycles; denaturing at 94 °C, 15 seconds, annealing at 60 °C, 30 seconds, and primer extension at 60 °C for 30 seconds), and a melt curve analysis from 65 °C-95 °C at 0.5 °C/second.

**Table 1:** Primer sequences for quantitative RT-PCR of rat enzymes and transporters involved in L-carnitine homeostasis

**Gene**	**Accession Number**	**Primers**	
		**Forward**	**Reverse**
β-actin	NM_031144	agcgtggctacagcttcacc	tgccacaggattccataccc
Octn1	NM_022270	catggctgtgcagactgg	gcaccatgtagccgatgg
Octn2	NM_019269	ggcgcaaccacagtatcc	ggggctttccagtcatcc
Octn3	NM_019723	gacaccgtgaacctgagc	ccatccaggcagttctcc
Cpt1b	NM_013200	cagccatgccaccaagatc	aagggccgcacagaatcc
Cpt2	NM_012930	gctccgaggcgtttctca	tggccgttgccagatagc
Bbh	NM_022629	acgatggggcagagtcc	ctggcctcctgagaaaagc
Tmlh	NM_133387	aatgtccctcccactcagg	tcggtatggcgatctaggg

Octn, organic cation/carnitine transporter; Cpt, carnitine palmitoyltransferase; Bbh, γ-butyrobetaine hydroxylase; Tmlh, trimethyllysine hydroxylase.

### Validation of Primers

Quantitative RT-PCR assays were initially optimized to give PCR efficiency between 1.9-2.1 (as determined by a 3-point standard curving using serial dilutions of control RNA with a slope range of −2.9 to −3.5) and a single melt-peak corresponding to the appropriate PCR product as verified by 2 % agarose gel electrophoresis. In addition, the amplification efficiency of each target and β-actin was determined by constructing a standard curve from the crossing point (C_T_) value and RNA concentration. The target genes and β-actin were then amplified using the same diluted samples. The ΔC_T_ values were calculated (i.e. the difference between the target gene C_T_ and β-actin C_T_). The slope from log RNA concentration versus ΔC_T_ was close to zero (<0.1). Only primers giving PCR amplification close to 100 % and relative efficiencies between the target and β-actin that were approximately equal were used in our experiment.

### Heart High Energy Phosphate Substrate Determination

The heart high energy phosphate substrate levels including creatine (Cr), creatine phosphate (CrP), ATP, ADP and AMP were measured with HPLC-UV method as described by Olkowski et al. [24]. The heart samples were homogenized in 0.7 M ice-cold perchloric acid (MW 100.46 D) with a final concentration 100 mg/mL. The homogenate was centrifuged at 12,000 rpm for 5 minutes. The supernatant was collected and neutralized with 2 M potassium hydroxide to bring pH near to 7.0. The supernatant was then filtered through a 0.45 μm filter (Nonsterile Syringe Filter Nylon, Chromatographic Specialties Inc. Brockville, Ontario, Canada) and 10 μL was injected onto a 3 μm Luna C18 (Phenomenex, Torrance, CA) column using gradient flow conditions. Two mobile phase components used included 20 mM potassium phosphate buffer (pH 7.0) and 100 % methanol. The gradient was 100 % phosphate buffer from 0–6.5 min, 100 % methanol from 6.5–12.5 min, and 100 % phosphate buffer from 12.5 to 25 min for column re-equilibration, which was sufficient to achieve stable baseline conditions. The high-energy phosphate substrates including CrP, Cr, ATP, ADP and AMP were monitored at 210 nm. The standard curve range was from 6.25-100 μg/mL and the limit of detection was 0.078 μg/mL for ATP and 0.312 μg/mL for ADP and AMP. Intra- and interassay accuracy and precision ranged from 4.2 % to 14.5 %.

#### Immunohistochemistry

Intestine and kidney from neonatal rat pups were rapidly harvested and fixed by immersion in 4 % paraformaldehyde. Paraffin imbedded sections (5 μm) were prepared by standard procedures and sections were incubated with Octn antibodies (1:100) (Alpha Diagnostic International) for 1 h followed by (H + L)-peroxidase-conjugated affinity-pure goat anti-rabbit IgG (1:500) for 30 min. Immunoreactions were visualized by using VIP Substrate kit for peroxidase (Vector Laboratories) according to manufacturer instructions. Non-specific binding was tested by using premixed control peptide with the respective Octn antibodies. For negative controls, only secondary antibody was applied to the slides.

### Statistical Analysis

All data are reported as mean ± SEM. One-way ANOVA with Tukey’s post hoc test was used for the comparisons between ages. A *P* < 0.05 was chosen as the level of statistical significance. Pearson's correlation coefficients were computed to quantify the association between heart L-carnitine concentration and heart Octn2 mRNA expression levels as well as serum L-carnitine concentration and kidney Octn2 mRNA expression.

## Results

**Figure 1:**
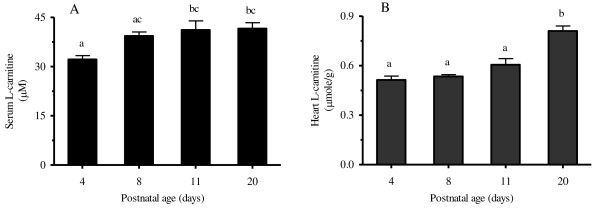
**Mean ± SEM free L-carnitine concentration in rat (A) serum and (B) heart tissue (n = 6) at postnatal day 4, 8, 11, and 20.** L-Carnitine concentration was determined using HPLC-UV as described in text. One-way ANOVA with Tukey’s post hoc test was used for the comparisons between ages and bars with the same letters indicate no significant difference, *P* < 0.05.

To examine the ontogenesis of critical components of the L-carnitine homeostasis pathway we conducted an mRNA expression and activity analysis of various components of the homeostasis pathway in different tissues of rat pups of different postnatal ages. Serum free L-carnitine concentrations (Figure 1A) increased significantly between postnatal day (PD) 4 and PD11 (PD11 L-carnitine concentrations were approximately 20 % higher than PD4) and stayed reasonably stable after PD11. Heart free L-carnitine concentrations (Figure 1B) were relatively similar at PD4, PD8 and PD11, but a significant increase was observed between PD11 and PD20 (heart L-carnitine concentrations were approximately 50 % greater at PD20 relative to PD4).

**Figure 2:**
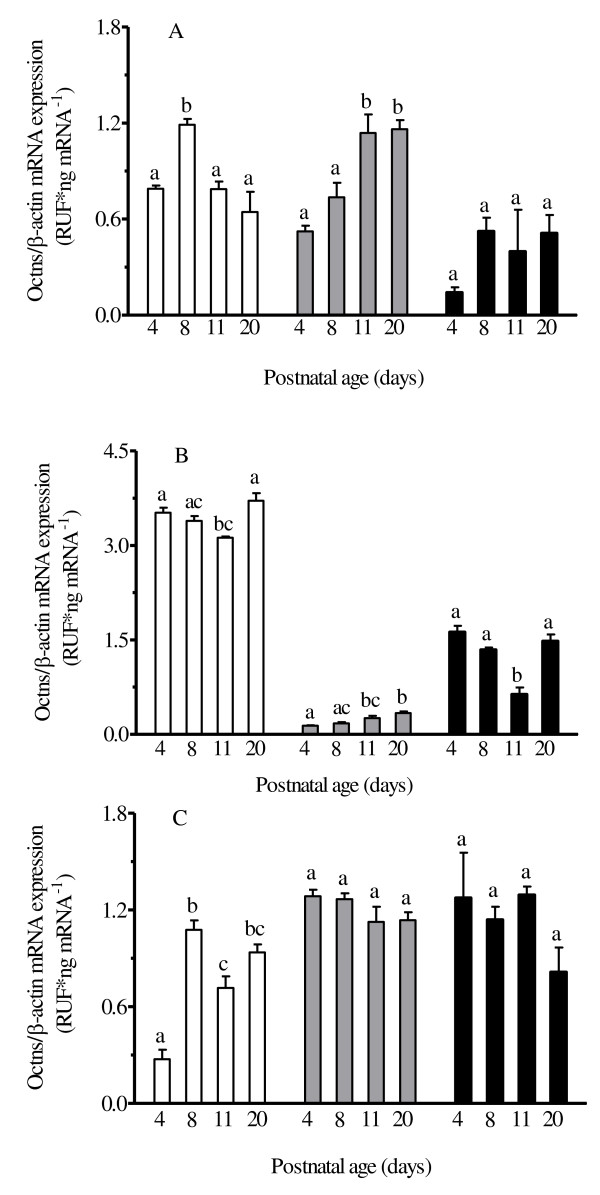
**Mean ± SEM organic cation/carnitine transporter (Octn) mRNA expression (Octn1 – white bar; Octn2 – light grey bar; Octn3 – dark grey bar) in rat (A) heart, (B) kidney, and (C) intestine at postnatal day 4, 8, 11, and 20 (n = 6).** mRNA expression was normalized to β-actin and fold difference (FD) was determined by using 2^-ΔΔCT^ method. One-way ANOVA with Tukey’s post hoc test was used for the comparisons between ages and bars with the same letters indicate no significant difference, *P* < 0.05.

**Figure 3:**
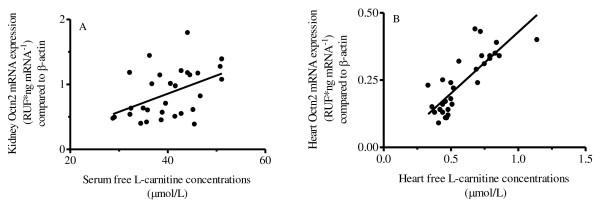
**(A) Correlation between rat kidney Octn2 mRNA expression and serum free L-carnitine concentration (Pearson’s r = 0.462).** (**B**) Correlation between heart Octn2 mRNA expression and heart free L-carnitine concentration (Pearson’s r = 0.823).

mRNA expression of key L-carnitine transporters, the Octns, were evaluated in the heart, kidney, and intestine by QRT-PCR. Generally, heart Octn1 mRNA expression (Figure 2A) remained constant, except at PD8, where expression was statistically higher than all other PD age groups. Heart Octn2 expression (Figure 2A) increased postnatally (approximately 100 % increase in expression at PD11 relative to PD4), although increases in expression after PD11 were not statistically significant. Although not statistically significant, heart Octn3 mRNA expression (Figure 2A) was lowest at PD4 while expression was approximately 200 % higher at PD8 and remained relatively constant thereafter. A strong (r = 0.823) and significant correlation (*P* < 0.05) (Figure 3B) was observed between heart free L-carnitine concentrations and heart Octn2 mRNA expression in rat pups at different postnatal age groups.

In the kidney, Octn1 and Octn3 mRNA expression (Figure 2B) was relative stable throughout postnatal development, although PD11 demonstrated a statistically significant decrease in expression relative to all other PD age groups. This consistent result between Octn1 and Octn3 likely has limited importance as it probably results from expected interindividual variation and the use of a destructive sampling strategy. Kidney Octn2 expression (Figure 2B) increased in the postnatal period, although increases in expression after PD11 were not statistically significant. Serum free L-carnitine concentrations were significantly correlated (r = 0.462) to kidney Octn2 mRNA expression (*P* < 0.05) (Figure 3A) in rat pups at different postnatal age groups.

In the intestine, Octn1 mRNA expression (Figure 2C) was low at PD4 but demonstrated a statistically significant increase (approximately 200 %) at PD8. Expression at PD20 was similar to PD8, although PD11 demonstrated a statistically significant decrease in expression relative to PD8 and PD20. Relative to Octn1 mRNA expression, intestinal Octn2 and Octn3 mRNA expression (Figure 2C) was high in the early postnatal period. Expression of Octn2 and Octn3 remained relatively constant through the remaining postnatal period.

**Figure 4:**
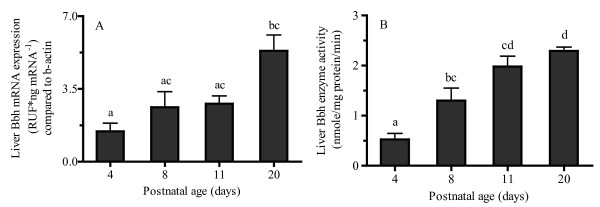
**Mean ± SEM rat liver gamma-butyrobetaine hydroxylase (Bbh) (A) mRNA expression and (B) activity at postnatal day 4, 8, 11, and 20 (n = 6).** mRNA expression was normalized to β-actin and fold difference (FD) was determined by using 2^-ΔΔCT^ method. Bbh activity was measured by quantifying the conversion of γ-butyrobetaine to L-carnitine by HPLC-UV. One-way ANOVA with Tukey’s post hoc test was used for the comparisons between ages and bars with the same letters indicate no significant difference, *P* < 0.05.

The postnatal increase in liver Bbh mRNA expression generally mirrored the increase in Bbh activity (Figure 4). Although not statistically significant, Bbh mRNA expression (Figure 4A) increased approximately 50 % between PD4 and PD8 with a statistically significant increase (more than 100 %) between PD8 and PD20. An approximately 400 % increase in liver Bbh activity (Figure 4B) was observed between PD4 and PD11 and activity remained relatively constant thereafter.

**Figure 5:**
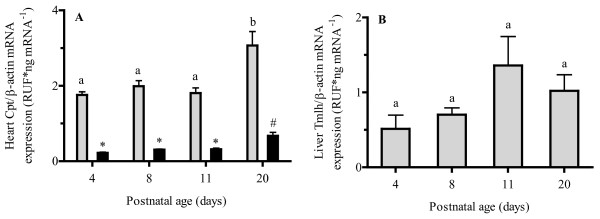
**Mean ± SEM mRNA expression of (A) heart carnitine palmitoyltransferase 1b (Cpt1b) (light grey bar) and carnitine palmitoyltransferase 2 (Cpt2) (black bar), and (B) hepatic trimethyllysine hydroxylase (Tmlh) in rat pups at postnatal day 4, 8, 11, and 20 (n = 6).** mRNA expression was normalized to β-actin and fold difference (FD) was determined by using 2^-ΔΔCT^ method. One-way ANOVA with Tukey’s post hoc test was used for the comparisons between ages and bars with the same letters (mRNA expression levels) or same symbol (activity levels) indicate no significant difference, *P* < 0.05.

**Figure 6:**
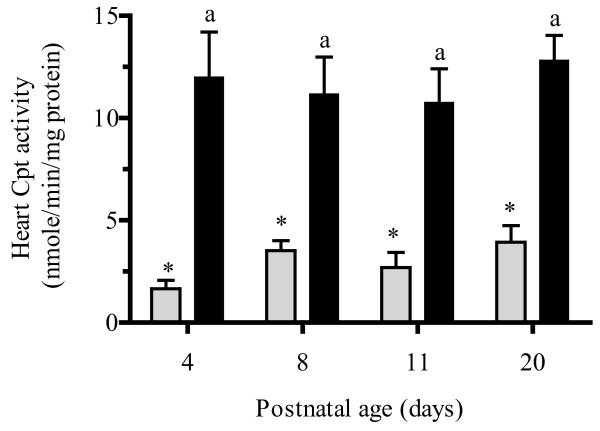
**Mean ± SEM heart carnitine palmitoyltransferase 1b (Cpt1b) (light grey bar) carnitine palmitoyltransferase 2 (Cpt2) (dark grey bar) activity in rat pups at postnatal day 4, 8, 11, and 20 (n = 6).** Heart Cpt enzyme activities were measured using the spectrophotometric method described by Bieber et al. [21]. One-way ANOVA with Tukey’s post hoc test was used for the comparisons between ages and bars with the same symbol (Cpt1) or same letters (Cpt2) indicate no significant difference, *P* < 0.05

**Figure 7:**
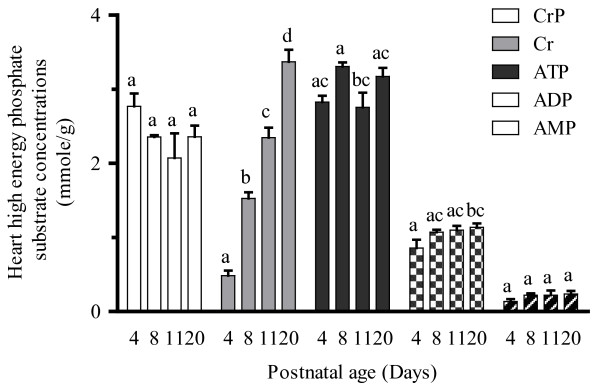
**Mean ± SEM heart high-energy phosphate substrate concentration in rat pups at postnatal day 4, 8, 11, and 20 (n = 6).** The heart high energy phosphate substrate levels including creatine (Cr), creatine phosphate (CrP), ATP, ADP and AMP were measured with HPLC-UV method as described by Olkowski et al. [24]. One-way ANOVA with Tukey’s post hoc test was used for the comparisons between ages and bars with the same symbol indicate no significant difference, *P* < 0.05. (Cr – creatine; CrP – creatine phosphate).

Heart Cpt1b and Cpt2 and liver Tmlh mRNA expression (Figure 5A and 5B) remained constant in the early postnatal period with significant increases in expression (approximately 50 %) occurring only between PD11 and PD20 for Cpt enzymes, while no change in Tmlh mRNA expression was observed with postnatal age. Despite this increase in Cpt mRNA expression in the late postnatal period, no significant differences in Cpt enzyme activity (Figure 6) were found at all examined postnatal ages. In addition, heart high energy phosphate concentrations (Figure 7) remained relatively constant through postnatal development. The heart concentrations of creatine (Figure 7) increased significantly during postnatal development. An almost 250 % increase in heart creatine concentrations was observed between PD4 and PD8 and a more than100% increase between PD8 and PD20. Creatine phosphate levels in postnatal cardiac tissue (Figure 7) remained relatively constant at all postnatal age groups.

Immunohistochemical staining with Octn antibodies was performed to determine Octn expression in kidney, intestine and heart *in situ*,. However, staining for Octn transporters in PD11 could not be distinguished from control and no further evaluations were conducted.

## Discussion

During postnatal development, a number of nutrient homeostasis pathways undergo ontogenesis to satisfy the nutrient requirements for growth and development. Although many of the developmental changes in these nutrient homeostasis pathways are genetically programmed, exogenous factors such as dietary components, environmental compounds and therapeutic drugs might affect their maturation. Our laboratory is interested in the developmental outcomes of interactions between drugs and nutrients that share the same absorption and/or disposition (i.e. distribution, elimination mechanisms) mechanisms. Our investigations began with L-carnitine homeostasis pathways and we systemically evaluated the ontogeny of key L-carnitine homeostasis pathways in the rat. Such information can help us to further understand the impact of exogenous factors on the maturation of nutrient homeostasis processes and the possible long-term consequences of drug-nutrient interactions during ontogeny.

In mammals, fatty acid oxidation becomes the main source of energy for many tissues with transition to extrauterine life [25]. Mitochondrial fatty acid utilization, though, requires a sufficient supply of L-carnitine to shuttle long-chain fatty acids across the mitochondrial membrane making them available for β-oxidation [2]. The maternal circulation supplies L-carnitine to the developing fetus. During late gestation, L-carnitine concentrations significantly increase in fetal tissues and this storage of L-carnitine assures adequate levels in the immediate postpartum period [9,26]. These tissue stores quickly become depleted due to the immaturity of many of the L-carnitine homeostasis mechanisms and maintenance of L-carnitine concentrations requires an exogenous source (i.e. milk) of L-carnitine [9]. In our study, postnatal increases in serum free L-carnitine were consistent with levels reported in the literature [27] and these increases correlated with maturation of a number of enzymes and transporter systems that critically determine L-carnitine levels in the body.

Lower serum free L-carnitine levels in the early postnatal period is, in part, due to the limited capacity for endogenous biosynthesis by the young neonate [5,19]. As noted in other studies, TLMH mRNA expression remained constant with postnatal development [6] and in agreement with other studies we found that hepatic γ-Bbh mRNA expression and activity was significantly lower at early postnatal development in rat pups [19,28]. Young neonates are highly dependent on exogenous sources of L-carnitine, which is usually supplied in sufficient amounts by the breast milk during nursing [29]. Interestingly, L-carnitine levels in the milk of nursing rat dams decrease significantly by mid-lactation [30,31]. Despite the reduced exogenous L-carnitine, serum free L-carnitine levels in rat pups increase with advancing age [27]. The maturation of hepatic γ-Bbh contributes to the postnatal increase in L-carnitine levels in the body. However, our study also suggests that maturation of other processes, namely gastrointestinal absorption and renal reabsorption of L-carnitine additionally contributed to the postnatal rise in serum L-carnitine.

Absorption of dietary sources of L-carnitine requires the function of several transporter systems expressed at the gastrointestinal epithelial barrier [25]. In our study expression of small intestinal Octn2 and Octn3 did not change with postnatal development, while Octn1 expression increased significantly between PD4 to PD8 remaining relatively constant after PD8. A previous study, which examined the postnatal maturation of the intestinal uptake L-carnitine, noted that Na^+^-dependent and Na^+^-independent intestinal uptake of L-carnitine was high in late gestation and in the newborn and significantly decreased between PD1 and PD15 [32]. Furthermore, mRNA expression of Octn2 demonstrated only a 20 % decrease between PD1 and PD15 in the jejunum, while ileal expression demonstrated a 100 % decrease between these two postnatal age groups [32]. In our study Octn2 mRNA expression was evaluated in the jejunum, and the constant expression of Octn2 and Octn3 is consistent with these findings. Octn2 and Octn3 mediates the Na^+^-dependent uptake of L-carnitine at the small intestine [11,33], and although not statistically significant, our data demonstrates that Octn2 expression did decrease at PD11 and PD20 and Octn3 at PD20, which might suggest a reduced ability to absorb L-carnitine with advancing postnatal age as previously reported [32].

Renal reabsorption of L-carnitine from the urinary filtrate plays a significant role in maintenance of L-carnitine levels in the body. Almost 95 % of the excreted L-carnitine is reabsorbed by transporters expressed in the proximal tubules of the kidney with Octn2 as the principal transporter involved in this process [12]. In our study renal Octn2 expression increased during postnatal development in the rat, which is consistent with the literature [12,14,34]. The increase in renal Octn2 expression correlated strongly with increases in serum L-carnitine levels suggesting that renal Octn2 plays a significant role in the postnatal pattern of serum L-carnitine development. Overall our data suggest the developmental changes in hepatic γ-Bbh expression, intestinal Octn1 expression, and renal Octn2 expression might systemically contribute to the postnatal increase in serum L-carnitine levels. However, the precise interconnections of these pathways and their overall contribution to L-carnitine homeostasis during development is not known and further studies are required to clarify their contributions.

The distribution of L-carnitine in the body is organ dependent with the highest concentration of L-carnitine in the heart [35]. In our study, heart L-carnitine levels increased during postnatal development and these increases were correlated with increased expression of Octn2 in the heart. L-Carnitine has a significant function in energy production in neonatal cardiac tissue due to its role in fatty oxidation and the reliance of neonatal hearts on fatty acids as the primary energy substrate [36]. The dramatic increase in fatty acid oxidation rates in early heart development after birth has been attributed to an increase in L-carnitine levels [36]. Although we observed a significant increase in L-carnitine levels in the heart with advancing age of the neonate, cardiac ATP levels remained constant through postnatal development. Interestingly, we found that creatine and ADP levels were ontogenically regulated during postnatal development. The significance of such developmental changes is not clear and requires investigation.

We also evaluated heart Cpt enzyme expression and activity due to the pivotal role of these enzymes in heart energy production. The postnatal increase in both Cpt1b and Cpt2 mRNA expression at postnatal day 20 are paralleled by the increases in heart L-carnitine concentrations. Indeed, Cpt1a and Cpt2 mRNA levels were increased by carnitine administration in cell culture systems [37]. Thus, the significant increase in heart L-carnitine levels at postnatal day 20 might account for the transcriptional enhancement of both Cpt1b and Cpt2. Despite these transcriptional increases in Cpt enzymes, we observed no significant changes in heart Cpt enzyme activity. Cpt enzyme activities have been reported to increase with increasing mitochondrial L-carnitine levels [38-40]. Unfortunately, L-carnitine levels in the whole heart tissue rather than in the mitochondria were measured in our study.

In conclusion, several L-carnitine homeostasis pathways underwent significant ontogenesis during postnatal development in the rat. However, the exact relationship between these pathways and their contribution to L-carnitine homeostasis during development is not completely known and further studies are required to clarify their contributions. Such a clarification is necessary to understand the impact of exogenous and endogenous factors on L-carnitine status during development. Nonetheless, this systematic evaluation of key pathways in the L-carnitine homeostasis pathway provides a basis from which we can conduct further evaluations regarding the effects of exogenous (i.e. drug) and endogenous factors (i.e. disease) on L-carnitine status during postnatal development and possible long-term consequences of any disturbance in the normal ontogeny of these pathways.

## Abbreviations

Bbh, γ-butyrobetaine hydroxylase (Bbh); Cpt, Carnitine palmitoyltransferase; Tmlh, Trimethyllysine hydroxylase; Octn, Organic cation/carnitine transporter; QRT-PCR, Quantitative reverse transcription-polymerase chain reaction, DTNB, 5,5’-dithio-bis (2-nitrobenzoic acid) (DTNB); PD, Postnatal day; Cr, Creatine (Cr); CrP, Creatine phosphate; ATP, Adenosine triphosphate; ADP, Adenosine diphosphate; AMP, Adenosine monophosphate.

## Competing interesting

The authors declare that they have no competing interest.

## Author’s contributions

All authors contributed substantially to the body of work and have read and approved the final submitted manuscript.
